# Physical activity and the risk of aseptic loosening after total hip arthroplasty: a case–control study from the Norwegian Arthroplasty Register

**DOI:** 10.1186/s12891-025-08865-9

**Published:** 2025-07-04

**Authors:** J. V. Nordbø, T. M. Straume-Næsheim, G. Hallan, A. M. Fenstad, E. A. Sivertsen, A. Årøen

**Affiliations:** 1 Department of Orthopedic Surgery, Akershus University Hospital, Lørenskog, Norway; 2 Institute of Clinical Medicine, Campus Ahus, University of Oslo, Oslo, Norway; 3 Department of Orthopedic Surgery, Haukeland University Hospital, Bergen, Norway; 4 Department of Clinical Medicine (K1), University of Bergen, Bergen, Norway; 5 Department of Orthopedic Surgery, Haukeland University Hospital, Bergen, Norway; 6 Department of Orthopedic Surgery, Lovisenberg Diaconal Hospital, Oslo, Norway; 7 Oslo Sports Trauma Research Center, The Norwegian School of Sport Sciences, Oslo, Norway

**Keywords:** Osteoarthritis, Total hip arthroplasty, Highly cross-linked polyethylene, Physical activity, Aseptic loosening, Wear

## Abstract

**Introduction:**

Previous studies have shown increased wear rate and aseptic loosening of total hip arthroplasty (THA) in patients with high physical activity levels. However, highly cross-linked polyethylene (HXLPE) has a reduced wear rate compared to conventional polyethylene. Thus, studies on the amount of physical activity in THA with HXLPE are needed. We aimed to determine if the level of physical activity in patients with THA containing HXLPE was associated with the risk of aseptic loosening of this implant.

**Methods:**

In this retrospective case–control study, we identified patients aged 40–75 years with a primary THA containing HXLPE registered in the Norwegian Arthroplasty Register (NAR) from 2005–2012 (*n* = 10,904). With a mean follow-up of 9.1 years, 176 patients were revised for aseptic loosening of their THA and invited into the study together with a sample of 856 patients with unrevised THA. Participants reported their physical activity level by answering the University of California, Los Angeles (UCLA) activity score at their best-remembered condition after the primary surgery. The peak physical activity levels were compared to the risk of being revised due to aseptic loosening in a multivariate logistic regression analysis.

**Results:**

Patients treated with revision surgery for aseptic loosening (*n* = 77 (44%)) had a lower retrospective peak level of physical activity before revision surgery compared to unrevised control patients (*n* = 429 (50%)); UCLA score median (IQR) 7 (5–8) vs. 8 (6–8). Adjusted logistic regression analyses of these UCLA scores on the risk of revision surgery due to aseptic loosening of the THA, showed that a higher level of physical activity was associated with a decreasing risk of revision surgery (OR 0.8, 95% CI 0.7–0.9, *P* = 0.001).

**Conclusion:**

A retrospectively self-reported level of physical activity at the patient`s best-remembered condition after primary THA was not associated with a higher risk of revision surgery due to aseptic loosening of the THA. Patients with THA should be encouraged to gain the health benefits of regular physical activity rather than restricting their activity level because of fear of implant wear and loosening.

**Supplementary Information:**

The online version contains supplementary material available at 10.1186/s12891-025-08865-9.

## Introduction

Physical activity is essential not only to improve quality of life, but a moderate to vigorous level of physical activity can reduce morbidity like cardiovascular disease [1]. Patients with end-stage osteoarthritis (OA) in the hip experience pain and joint stiffness, which can be successfully treated by total hip arthroplasty (THA) [2, 3]. In a recent study, we showed that middle-aged people with THA were more physically active than a control group representing a normal population [4]. Low-impact sports like walking or cycling are the most common activity after THA [5]. Due to an expected increase in younger patients undergoing THA in the years to come and the known health-enhancing benefits of physical activity, it is essential for patients with THA to perform physical activity as they want [6–8].

Known barriers for patients to engage in high-level physical activity after THA are patients` fear of damaging their THA and caution from health care professionals [9, 10]. The most cited risk caused by the return to sport after THA is the aseptic loosening of the implant [10, 11]. The theory is that particle debris made from the articulation of the THA introduces an inflammatory process that leads to osteolysis and loosening of the implant [12]. According to this theory, increased use of the artificial joint will lead to more particle debris and, subsequently, a higher risk of loosening the implant. With the introduction of highly cross-linked polyethylene (HXLPE) in THA, the wear rate is reduced [13], but there is a lack of studies that show how it interferes with high levels of physical activity and the risk of aseptic loosening [10]. This study aimed to determine if patients revised for aseptic loosening of the THA had higher levels of physical activity before revision surgery than unrevised control patients.

## Methods

### Study design and data collection

In this retrospective case–control study, the cases constitute patients with THA revised for aseptic loosening, and the controls constitute patients with an unrevised primary THA. We identified patients in the Norwegian Arthroplasty Register (NAR) who had a unilateral primary THA with articulations made of metal or ceramic femoral head on an HXLPE counter-surface from 2005 to 2012. HXLPE was introduced in Norway in 2005, and we chose an inclusion period of seven years to obtain a sufficient sample size of THAs containing HXLPE. We selected patients aged 40–75 years at the time of surgery due to their expected higher physical activity levels than older patients [14]. All hospitals in Norway report primary THA to the NAR with a completeness of 97% and revision surgery with a completeness of 91% [15].

By 31 December 2019, with a mean follow-up of 9.1 years (7.0 to 14.3 years), we identified 10,541 THAs eligible for the study. In this sample, 176 patients were revised for aseptic loosening of their THA, constituting the study`s cases. From the same sample, we randomly selected a control group (*n* = 856) of the unrevised THAs that was five times larger than the cases to increase statistical power. The study coordinator invited the cases and controls to participate through a questionnaire sent by mail in December 2020, followed by two reminders. A secretary plotted the answers to a digital database, and the study coordinator controlled the data. The study is reported according to the STROBE guidelines [16].

### Variables

**Fig. 1:**
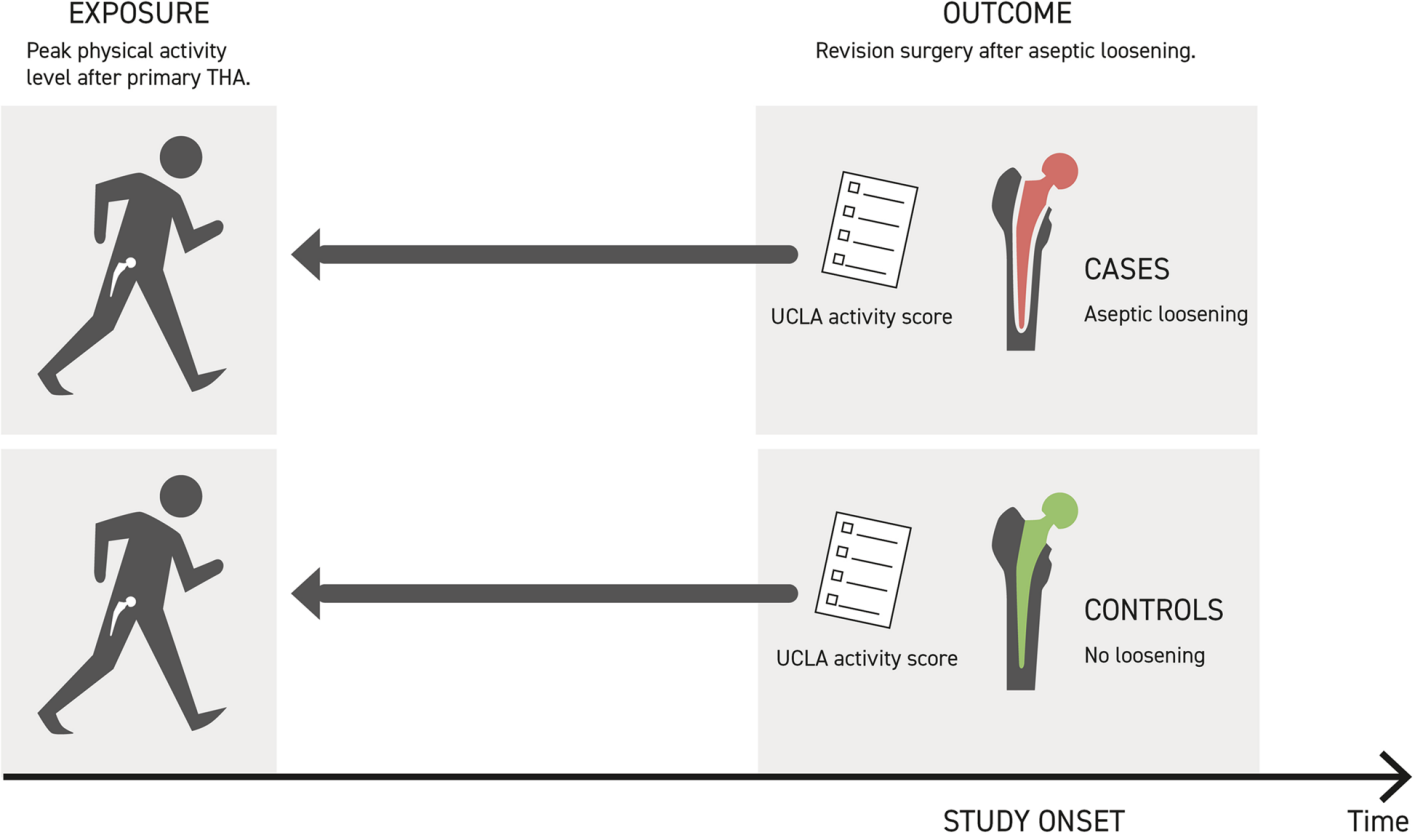
The figure illustrates the case–control study design. Both cases and controls reported their physical activity levels by answering the UCLA activity score retrospectively at their best condition after the primary surgery. This level of physical activity constituted the exposure for the identified outcome, revision surgery for aseptic loosening of THA (Illustration by Hellevik studio)

Participants reported their physical activity levels by answering the previously validated University of California, Los Angeles (UCLA) activity score (Appendix) [17]. The UCLA score is a 10-level scale ranging from wholly inactive (level 1) to highly physically active (level 10). Due to the lack of prospectively collected physical activity levels after the primary THA, all patients were asked to report their physical activity levels retrospectively at their best condition after primary surgery (Fig. 1). This level of physical activity constituted the primary exposure for the identified outcome, revision surgery due to aseptic loosening of the THA.

Secondary exposure measures, EQ-5D-5L, EQ VAS, and Hip Disability and Osteoarthritis Outcome Score (HOOS), were collected similarly retrospectively at the patients best condition after primary surgery (Appendix). The EQ-5D-5L is an extensively validated generic 5 item questionnaire (mobility, self-care, usual activities, pain/discomfort and anxiety/depression) concerning general health [18], which is calculated into a -0.59 (worst) to 1.0 (best) index. To calculate the EQ-5D index values from EQ-5D-5L, we used scores from the EQ-5D-3L-UK value set through a crosswalk algorithm [19, 20]. The EQ VAS is a vertical visual analogue scale ranging from 0 (worst health) to 100 (best health), where patients rate their general health. The HOOS is a validated hip-specific questionnaire with the subgroups` symptoms, pain, activities of daily living, sports and quality of life [21]. To calculate the mean values of the HOOS subgroups, we used the transformation to a 0 (worst) to 100 (best) scale presented by Roos et al. [22]. Missing values were substituted with the average value for the subgroup unless more than two values of a subgroup were omitted.

The NAR collects baseline characteristics of all primary THAs at the time of surgery, which includes information on the patient`s age, sex, date of the operation, American Society of Anesthesiologists (ASA) classification, indication for surgery, type of surgical procedure, surgical approach, and fixation method.

### Sample size

The cohort`s sample size is based on a meaningful difference in the UCLA score. SooHoo [23] defined the minimal clinically important difference in UCLA score as 0.92, and Lubbeke [24] defined the standard deviation (SD) as 2.0. This estimate requires 148 patients, 74 in each group, to obtain 80% statistical power with a 5% significance level for an independent samples t-test.

### Statistical analysis

Descriptive statistics were presented for cases and controls, and respondents and non-respondents, as means and standard deviations for continuous variables, compared with independent-samples t-test, and numbers and percentages for categorical variables, compared with chi-squared test.

The UCLA score, EQ-5D index, and HOOS scores were not expected to be normally distributed; thus, we presented data with medians and interquartile range and analyzed between-group differences with the nonparametric Mann–Whitney U test. However, means and standard deviations were calculated to consider the variance in the scores and compare them with the current literature.

We used a logistic regression model to analyze the association of physical activity in the best condition after primary THA with the chance of revision surgery due to aseptic loosening. The association was adjusted for possible confounders such as age, sex, ASA class, surgical approach, and fixation method. The assumptions of the regression model were checked for linear effects and interactions on the log-odds scale. The model fit was considered according to the Akaike information criterion (AIC) and the Bayesian information criteria (BIC).

The correlation between physical activity levels after primary THA and time to revision due to aseptic implant loosening was visualized in a scatterplot and analyzed by a linear regression model.

Missing values on the primary exposure were checked among responders. With missing values > 10%, we would check if the values were missing completely at random (MCAR) or missing at random (MAR), and if so, conduct multiple imputation (MI) on the missing values. Missing values < 10% were considered not to influence the result significantly; in this case, MI was deemed unnecessary.

## Results

**Fig. 2:**
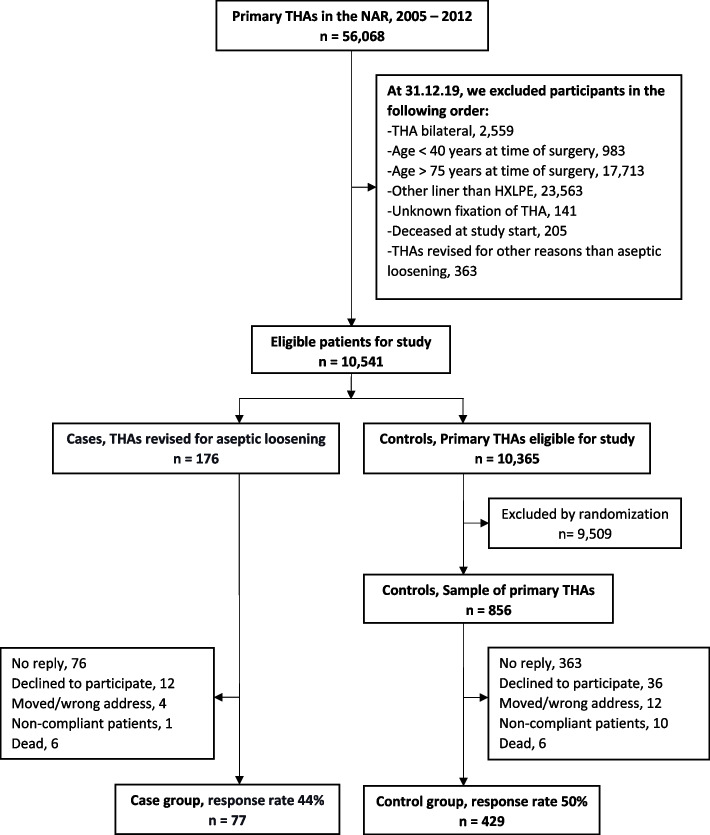
Flowchart of the selection of eligible patients from the Norwegian Arthroplasty Register (NAR) and the inclusion process. THA = Total hip arthroplasty, HXLPE = Highly cross-linked polyethylene

77 (44%) of the revised THA cases and 429 (50%) of the unrevised THA controls responded the questionnaire and were included into the study. The flowchart of Fig. 2 shows the selection process.

### Respondents vs. non-respondents

Both cases and controls were slightly younger and had fewer women than the non-respondents. In addition, the responding controls were healthier according to the ASA class, while the cases had a similar ASA class between the respondents and non-respondents. Regarding the hip-specific characteristics, both groups were similar regarding indication for surgery, surgical approach and fixation method between respondents and non-respondents (Appendix Tables 4 and 5).

### Cases vs. controls

The cases and controls were similar in age, sex, ASA class, indication for surgery and surgical approach. However, the cases had more cemented fixations (Table 1).

**Table 1:** Comparison of patient characteristics by cases, THAs revised for aseptic loosening, and controls, primary THAs. Values are counted (%) unless otherwise specified

Characteristic	Cases *n*=77	Controls *n*=429	Difference between groups (95% CI)	p
Mean age^a^ (SD)	61.0 (8)	62.0 (7)	-1.0 (-2.8 to 0.8)	0.27^b^
Female sex	41 (53)	247 (58)	-5% (-16 to 8)	0.48^c^
ASA-score				
ASA 1	21 (27)	154 (36)	-9% (-20 to 23)	0.14^c^
ASA 2	46 (60)	240 (56)	4% (-8 to 16)	0.54^c^
ASA 3+	8 (10)	29 (7)	3% (-4 to 11)	0.26^c^
Not reported	2 (3)	6 (1)	1% (-3 to 5)	0.44^c^
Indication for surgery				
Osteoarthritis	61 (79)	348 (81)	-2% (-12 to 8)	0.70^c^
Surg. approach				
Anterior (Smith-Petersen)	9 (12)	42 (10)	2% (-6 to 10)	0.61^c^
Anterolateral	11 (14)	59 (14)	0 (-8 to 9)	0.90^c^
Lateral	37 (48)	195 (45)	3% (-10 to 15)	0.67^c^
Posterior	19 (25)	121 (28)	-3% (-14 to 7)	0.52^c^
Not reported	1 (1)	12 (3)	-2% (-5 to 1)	0.39^c^
Fixation method				
Cemented	21 (27)	66 (15)	12% (1 to 22)	0.01^c^
Uncemented	19 (25)	152 (36)	-11% (-21 to 0)	0.07^c^
Hybrid	1 (1)	13 (3)	-2% (-5 to 1)	0.39^c^
Reversed hybrid	36 (47)	198 (46)	1% (-12 to 13)	0.92^c^

*THA* Total hip arthroplasty, *CI* Confidence interval, *ASA* American Society of Anesthesiologists

^a^Age at time of surgery

^b^Independent-samples t-test

^c^Chi-squared test

### Primary exposure measure

**Fig. 3:**
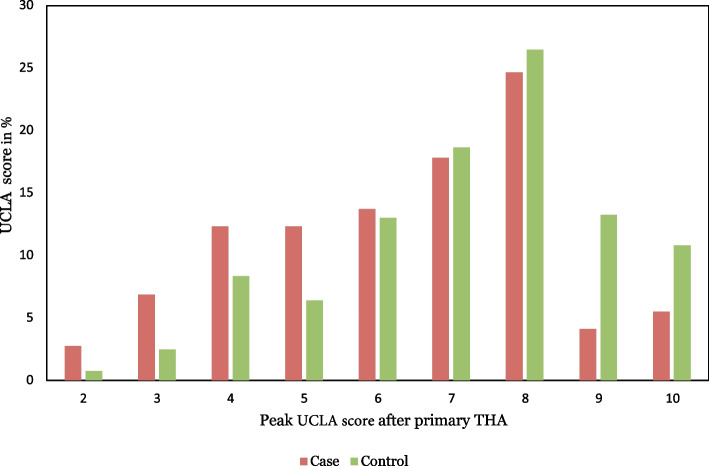
Distribution of retrospective peak UCLA scores after primary THA in % by cases and controls

The distribution of the peak UCLA scores is shown in Fig. 3. The retrospectively measured peak UCLA score after primary THA was lower in the cases (*n* = 73), median (IQR) 7 (5–8), compared to the unrevised controls (*n* = 408), median (IQR) 8 (6–8) (*p* = 0.001).

### Secondary exposure measures

**Fig. 4:**
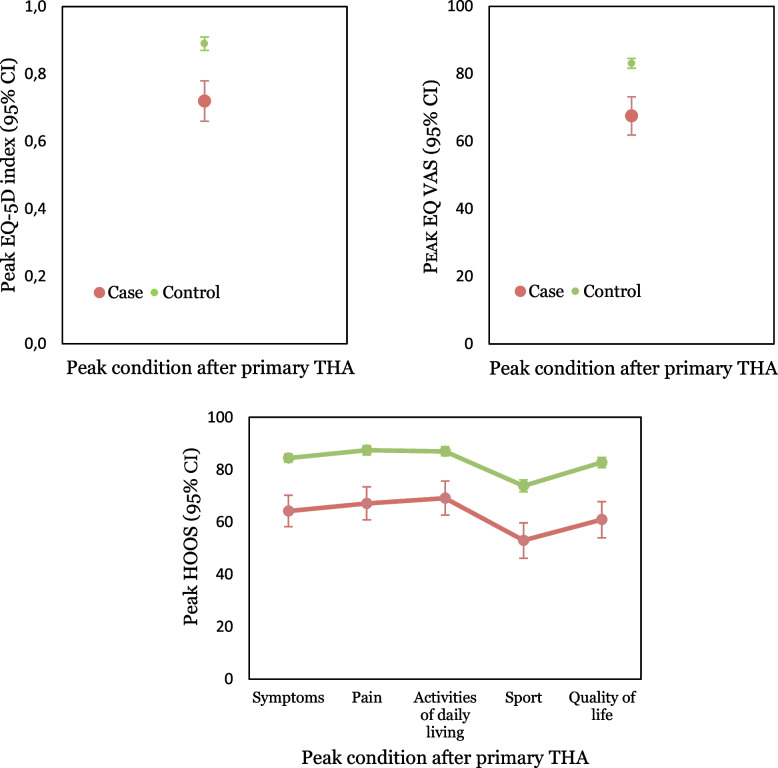
The distribution of retrospective peak EQ VAS, EQ-5D index, and all subdomains of HOOS after primary THA by cases and controls, reported with means and 95% CI

The peak EQ-5D index, EQ VAS, and all subdomains of HOOS were lower in the cases compared to controls, measured retrospectively after the primary THA (Table 2, Fig. 4).

**Table 2:** Retrospective peak primary and secondary exposure measures by cases and controls

Peak exposure measure	Cases			Controls			Cases vs Controls
	**n**	**Median (IQR)**	**Mean (SD)**	**n**	**Median (IQR)**	**Mean (SD)**	**p**^a^
UCLA score	73	7 (5–8)	6.3 (2.0)	408	8 (6–8)	7.2 (1.9)	0.001
EQ-5D index	66	0.8 (0.6–0.9)	0.7 (0.3)	404	1.0 (0.8–1.0)	0.9 (0.2)	<0.001
EQ-VAS	68	71 (50–88)	68 (23)	397	90 (78–91)	83 (15)	<0.001
HOOS symptoms	66	70 (50–85)	64.2 (24.5)	411	90 (75–100)	84.4 (16.4)	<0.001
HOOS pain	62	71 (50–89)	67.2 (24.8)	396	95 (83–100)	87.4 (17.6)	<0.001
HOOS activities of daily living	62	75 (56–92)	69.1 (25.8)	395	94 (81–100)	87.0 (17.4)	<0.001
HOOS sport	67	50 (31–75)	53.0 (27.5)	397	81 (56–94)	73.8 (23.2)	<0.001
HOOS quality of life	67	69 (38–81)	60.9 (28.2)	406	88 (75–100)	82.8 (19.9)	<0.001

*Cases* THAs revised for aseptic loosening, *Controls* unrevised THAs, *UCLA score* The University of California, Los Angeles activity score, *VAS* Visual analogue scale, *HOOS* Hip disability and Osteoarthritis Outcome Score with five subscores

^a^Mann-Whitney U test

### Physical activity and time to revision for aseptic loosening

**Fig. 5:**
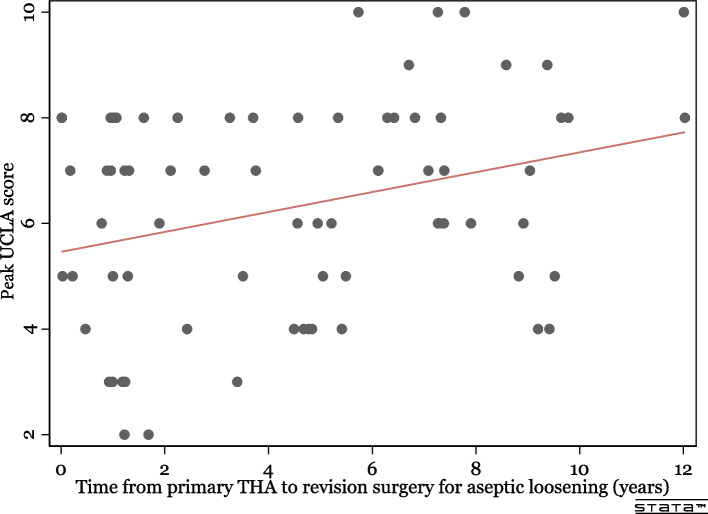
Patients treated with revision surgery for aseptic loosening of their THA had a linear increase of peak physical activity level before revision surgery at an extended time from primary surgery. The linear regression line shows a linear effect with an increase of 0.2 points of UCLA score for every year to revision, *p* = 0.01. The result did not change after adjusting for age, sex, ASA class**,** surgical approach, and fixation method

The time from primary THA to revision surgery for aseptic loosening ranged from 50 days to 12 years, with a mean of 4.5 years. We compared the measured physical activity levels after primary THA with time to revision and found a linear increase in physical activity at a longer time to revision (Fig. 5). Because of this finding, we ranged the cases based on time to revision and stratified them into quartiles. We did separate analyses on the first, early, and last, late quartile *n* = 19 (25%), constituting cases from 50 days to 1.2 years and 7.3 to 12 years from primary to revision surgery. The early quartile had a retrospective UCLA score of median (IQR) 6 (3–8), and the late quartile had 7 (6–8).

### Physical activity and the risk of aseptic loosening after THA

Logistic regression analyses of the retrospective physical activity levels on the risk of revision surgery due to aseptic loosening of the THA showed that higher physical activity was associated with a decreasing risk of revision surgery (OR 0.8, 95% CI 0.7–0.9. *P* < 0.001). The result did not change after adjustment for possible confounders, though cemented fixation was associated with a higher risk of revision surgery. We found a similar result when analyzing the early revision quartile. In logistic regression analyses of the retrospectively physical activity level in the late revision quartile compared to the unrevised controls, physical activity had no association with the aseptic loosening of the implant. The logistic regression analyses are presented in Table 3.

**Table 3:** Association of physical activity and the risk of aseptic loosening after Total Hip Arthroplasty

	**Controls**	**Cases**			
		**Unadjusted**		**Adjusted**^a^	
	**OR (95% CI)**	**OR (95% CI)**	**p**^b^	**OR (95% CI)**	**p**^b^
Model 1	1	0.8 (0.7 to 0.9)	0.00	0.8 (0.7 to 0.9)	0.00
Model 2	1	0.7 (0.5 to 0.8)	0.00	0.7 (0.5 to 0.9)	0.01
Model 3	1	0.9 (0.7 to 1.2)	0.55	1.0 (0.8 to 1.3)	0.97

^**a**^Adjusted for age, sex, ASA class**,** surgical approach, fixation method

^b^Logistic regression analyses

Model 1: All cases (*n* = 73) vs controls (*n* = 408)

Model 2: Early quartile (the first 19 THAs revised from aseptic loosening, from 50 days to 1.2 years after primary THA) vs controls (*n* = 408)

Model 3: Late quartile (the last 19 THAs revised from aseptic loosening, from 7.3 to 12 years after primary THA) vs controls (*n* = 408)

*OR* odds ratio. Also, see Table 1 for abbreviations

### Sensitivity analysis

With only 5% missing values on the retrospectively reported physical activity levels in both cases (4/77) and controls (21/429), MI on missing values was considered unnecessary.

Considering the assumptions of the logistic regression model, our observations were independent patients with unilateral THA from a national sample. The OR from the regression analyses was linear on the log odds scale. A cubic spline model with four knots had a lower model fit according to AIC and BIC. There was no significant interaction between the measured physical activity level and age, sex, or ASA class, except for physical activity and surgical approach. This interaction did not change the results from the primary analysis. Additionally, the model fit according to AIC and BIC was weaker after introducing this interaction.

## Discussion

### Main results

Patients revised for aseptic loosening of their THA reported a lower peak physical activity level before the revision surgery than patients still having their primary THA. The peak physical activity level before revision surgery was higher the longer these patients lived with their primary THA. However, neither the cases with extended time to revision (7.3 – 12 years) reported higher physical activity levels than controls with unrevised THA. Except for cemented THA fixation, the baseline data did not reveal any predictors for aseptic loosening. We found no association between a high level of physical activity and the risk of revision surgery due to aseptic loosening of the THA.

### Context within current literature

In a systematic review by Mooiweer on physical activity from one year and forward after THA, most articles reported mean UCLA scores between 5.5 and 7, which is comparable to our results [5]. The UCLA score is validated to be a useful measure of physical activity on a group level. Still, it has shown substantial variability on an individual level, with patients often overestimating their scores compared to investigators [17, 25]. This highlights a potential limitation in the data, as self-reported physical activity is generally known to be overestimated compared to objectively measured physical activity [26]. However, the simplicity of the scale measuring the amount of physical activity, and not only what the patient can do, makes it suitable for studies exploring physical activity in large groups.

We found no association between a high physical activity level and the risk of aseptic loosening of THAs containing HXLPE, with a minimum follow-up of 7 years. This is in line with the current literature. Streck et al. found no higher revision rates in THA patients with a high level of physical activity compared to a low level at a minimum of 2 years of follow-up [27]. Ennis et al. showed a similar result at a minimum 5-year follow-up [28]. Although these results seem uncontroversial based on the reduced wear rate provided by HXLPE, they contrast the known association between high physical activity levels and the aseptic loosening of THAs using conventional polyethylene. Thus, our results add knowledge to the recommendations on the amount of physical activity after THA [29]. An even more offensive interpretation would be to argue that physical activity prevents implants from aseptic loosening due to improved peri-prosthetic bone quality [30]. However, the retrospective observational design of this study cannot draw such conclusions; instead, it supports the hypothesis for future research.

Besides the lower peak physical activity levels before revision surgery, peak HOOS subscores, EQ-5D index, and EQ-VAS were low in the revised group compared to the unrevised and below previously published patient acceptable symptom state (PASS) values after THA [31, 32]. Thus, some cases did not seem to reach a sufficient reduction in pain or improvement in hip function after the index surgery. This might help us understand the low peak physical activity levels in cases that occur quickly from primary to revision surgery. It is unlikely that a well-fixed implant will fail in the early recovery phase, and these cases were more likely a result of failed primary fixation [33]. Additionally, confounders like sex, age, ASA class, surgical approach or fixation method did not influence the association between physical activity and time to revision.

We found a higher rate of all-cemented THA in the revision cases compared to controls. This association is seen in patients < 70 years, where cemented femoral stems are more associated with aseptic loosening than uncemented [34]. However, in a systematic review, Prock-Gibbs et al. found similar rates of osteolysis in cemented and uncemented THAs [13]. Though cemented THA were associated with a higher risk of aseptic loosening in our study, it did not influence the result. The direct anterior approach (DAA) showed an interaction with physical activity on increased risk of aseptic loosening. However, only 9 cases were operated through the DAA in our study. Due to the few observations, it isn’t possible to conclude the causal relationship between DAA and physical activity on the risk of aseptic loosening. Additionally, bringing this interaction into the logistic regression model of physical activity on the risk of aseptic loosening made the model less balanced, according to AIC and BIC [35]. Thus, omitting the interaction of DAA and physical activity from the model described our data better.

### Strengths and clinical applicability

We identified the study participants in the NAR, which makes our study sample representative of the average THA patient in Norway. The relatively low response rate was disappointing. However, we did have baseline data on the non-respondents from the NAR; this helped us to understand the respondents' representativeness. Additionally, only 5% of the observations of physical activity levels were missing among the responding cases and controls. We, therefore, considered the regression analyses of the results robust, with no need for further sensitivity analyses with imputation methods.

The average follow-up time of 9.1 years for THA patients with HXLPE was the longest possible in Norway at the study onset. HXLPE was introduced in Norway in 2005, and an inclusion period until 2012 was necessary to achieve a sufficient sample size. The long follow-up time of THA cases with HXLPE on physical activity and the risk of aseptic loosening of the implant is also unique in the international literature, with only a few publications available [27, 28].

Though this is a retrospective case–control study that is not fit for conclusions on causal relationships, the result of no association between physical activity and aseptic loosening of THA is intuitive due to reduced wear of HXLPE liners [13]. According to reduced wear in HXLPE liners, the use of UCLA score in the recent literature evaluates THA surgery rather than questioning the wear rate [25]. Thus, considering the benefits of physical activity [1], patients with THA should be recommended to perform physical activity rather than being restricted [8].

### Limitations and future perspectives

Since our study sample missed PROMs before revision surgery of the THA, a case–control design was the only option. Recall bias is a known challenge in this design, where the cases might have a “bad” experience of the revision surgery, not shared by the controls, which might influence their memory. To minimize the recall bias, we tried to simplify the question by asking about their peak physical activity level. By this phrasing, we asked of a state, hopefully, more accessible to remember than a specific time point.

We aimed to find a possible association between patients with high levels of physical activity and aseptic loosening of the THA. Since the characteristics of the respondents (cases and controls) were similar in age, gender, and ASA class, the selection bias made by the low response rate probably had a minor influence on the association between physical activity and aseptic loosening. However, the non-respondents were older and had a higher percentage of females. According to the ASA class, the non-respondents in the control group had more comorbidity than the respondents. Thus, it is reasonable to believe that patients who responded (both cases and controls) were more physically active than non-respondents. Additionally, we selected patients aged 40–75 years at the time of surgery, making the study sample younger than the average patient with a primary THA [36].

The observational study design is susceptible to confounding bias. Although regression analyses and examinations of interactions between variables are utilized, combinations of variables may constitute a potential confounding factor. Additionally, unmeasured confounding variables could influence the result.

The undefined criteria of aseptic loosening of the components is a source of bias. In the NAR, surgeons report the reason for revision surgery based on preoperative measures and intraoperative findings. Early cases of aseptic loosening were more likely failed fixations rather than true aseptic loosening. This is not possible to confirm in the present data. Additionally, we measured physical activity with a PROM. Though the scale is validated to report the patients' current physical activity level, we used it retrospectively despite the risk of recall bias. As mentioned, these measures are probably overestimated but suitable for studies in large groups [26].

The retrospective case–control design of this study is suitable for emphasizing our hypothesis. However, the results must be confirmed on prospectively collected physical activity levels in patients with THA. Such full-scale data collection was introduced in 2017 in the NAR, with pre-and post-THA measures of the UCLA activity score [36]. This enables longitudinally designed studies on physical activity levels in THA patients on the risk of revision surgery. Given the low wear rate of HXLPE, the association of physical activity with the aseptic loosening of THAs should be analyzed in the future with a longer follow-up time.

## Conclusions

At a mean follow-up of 9.1 years, patients revised for aseptic loosening of their THA did not remember to have a higher level of physical activity before the revision surgery, compared to patients still having their primary THA. Physical activity was not associated with an increased risk of revision surgery due to aseptic loosening of the THA. This finding supports the hypothesis that reduced wear rate in THAs with HXLPE makes the THA more resistant to a high level of physical activity. This result should be confirmed with prospectively collected physical activity levels in patients with THA at a longer follow-up. Our study implies that patients with THA should be encouraged to gain the health benefits of regular physical activity rather than restricting their activity level because of fear of implant wear and loosening.

## Supplementary Information

Supplementary Material 1.

## Data Availability

The datasets used in this study are available from the corresponding author upon reasonable request.

## Appendix

**Table 4:** Comparison of patient characteristics by respondents and non-respondents in the case group, THAs revised for aseptic loosening. Values are counted (%) unless otherwise specified

Characteristic	Respondents *n*=77	Non – respondents *n*=99	Difference between groups (95% CI)	p
Mean age^a^ (SD)	61.0 (8)	64.1 (8)	-3.1 (-5.5 to -0.8)	0.01^c^
Female sex	41 (53)	68 (69)	-16% (-30 to -1)	0.04^d^
ASA-score				
ASA 1	21 (27)	27 (27)	0% (-13 to 13)	1.00^d^
ASA 2	46 (60)	54 (55)	5% (-10 to 20)	0.49^d^
ASA 3+	8 (10)	15 (15)	-5% (-15 to 5)	0.35^d^
Not reported	2 (3)	3 (3)	0% (-5 to 5)	0.86^d^
Indication for surgery				
Osteoarthritis	61 (79)	80 (81)	-2% (-14 to 10)	0.79^d^
Hip dysplasia	6 (8)	7 (7)	1% (-7 to 9)	0.86^d^
Acute femoral neck fractures	1 (1)	3 (3)	-2% (-6 to 2)	0.45^d^
Other^b^	9 (12)	9 (9)	3% (-7 to 12)	0.57^d^
Surg. approach				
Anterior (Smith- Petersen)	9 (12)	12 (12)	0% (-10 to 9)	0.93^d^
Anterolateral	11 (14)	18 (18)	-4% (-15 to 7)	0.49^d^
Lateral	37 (48)	50 (51)	-3% (-17 to 12)	0.75^d^
Posterior	19 (25)	17 (17)	8% (-5 to 20)	0.22^d^
Not reported	1 (1)	2 (2)	-1% (-4 to 30)	0.71^d^
Fixation method				
Cemented	21 (27)	22 (22)	5% (-8 to 18)	0.44^d^
Uncemented	19 (25)	36 (36)	-11% (-25 to 2)	0.10^d^
Hybrid	1 (1)	1 (1)	0% (-3 to 3)	0.86^d^
Reversed hybrid	36 (47)	40 (41)	6% (-9 to 21)	0.40^d^

^a^Age at time of surgery

^b^Other indications includes femoral neck fracture, Rheumatoid arthritis, Bechterew`s disease, Calve-Legg-Perthes disease, proximal epiphysiolysis of the femur and other indications

^c^Independent-samples t-test

^d^Chi-squared test

See Table 1 for abbreviations

**Table 5:** Comparison of patient characteristics by respondents and non-respondents in the control group, THAs revised for aseptic loosening. Values are counted (%) unless otherwise specified

Characteristic	Respondents *n*=429	Non – respondents *n*=427	Difference between groups (95% CI)	p
Mean age^a^ (SD)	62.0 (7.4)	63.5 (8.5)	–1.5 (–2.6 to –0.4)^**b**^	0.01^c^
Female sex	247 (58)	282 (66)	–8% (–15 to –2)^**c**^	0.01^d^
ASA-score				
ASA 1	154 (36)	89 (21)	15% (11 to 26)^**c**^	0.00^d^
ASA 2	240 (56)	277 (65)	–9% (–16 to –3)^**c**^	0.01^d^
ASA 3+	29 (7)	56 (13)	–6% (–10 to –2)^**c**^	0.00^d^
Not reported	6 (1)	5 (1)	0% (–3 to 3)^**c**^	0.77^d^
Indication for surgery				
Osteoarthritis	348 (81)	331 (78)	3% (–2 to 9)^**c**^	0.19^d^
Hip dysplasia	44 (10)	39 (9)	1% (–3 to 5)^**c**^	0.58^d^
Acute femoral neck fractures	7 (2)	14 (3)	–1% (–4 to 1)^**c**^	0.12^d^
Other^b^	30 (7)	43 (10)	–3% (–7 to 1)^**c**^	0.11^d^
Surg. Approach				
Anterior (Smith-Petersen)	42 (10)	37 (9)	1% (–3 to 5)^**c**^	0.57^d^
Anterolateral	59 (14)	40 (9)	5% (1 to 9)^**c**^	0.05^d^
Lateral	195 (45)	201 (47)	–2% (–8 to 5)^**c**^	0.64^d^
Posterior	121 (28)	144 (34)	–6% (–12 to 1)^**c**^	0.08^d^
Not reported	12 (3)	5 (1)	2% (–1 to 3)^**c**^	0.09^d^
Fixation method				
Cemented	66 (15)	92 (21)	–6% (–11 to –1)^**c**^	0.02^d^
Uncemented	152 (35)	145 (34)	1% (–5 to 8)^**c**^	0.65^d^
Hybrid	13 (3)	3 (1)	2% (1 to 4)^**c**^	0.01^d^
Reversed hybrid	198 (46)	187 (44)	2% (–4 to 9)^**c**^	0.49^d^

^a^Age at time of surgery

^b^Other indications includes femoral neck fracture, Rheumatoid arthritis, Bechterew`s disease, Calve-Legg-Perthes disease, proximal epiphysiolysis of the femur and other indications

^c^Independent-samples t-test

^d^Chi-squared test

See Table 1 for abbreviations

## Abbreviations

AIC: Akaike information criterion
ASA class: American Society of Anesthesiologists classification
BIC: Bayesian information criteria
CI: Confidence interval
DAA: Direct anterior approach
EQ-5D-5L: Five-level-Euro-Qol five-dimension
EQ-VAS: EQ-5D visual analog scale
HOOS: Hip Disability and Osteoarthritis Outcome Score
HXLPE: Highly cross-linked polyethylene
IQR: Interquartile range
MAR: Missing at random
MCAR: Missing completely at random
MI: Multiple imputation
NAR: Norwegian Arthroplasty Register
OA: Osteoarthritis
OR: Odds ratio
PASS: Patient acceptable symptom state
PROM: Patient reported outcome measure
SD: Standard deviation
THA: Total hip arthroplasty
UCLA score: University of California, Los Angeles activity score
