# Supplementary material for: Physical activity and the risk of aseptic loosening after total hip arthroplasty: a case–control study from the Norwegian Arthroplasty Register
Source: BMC Musculoskelet Disord. 2025 Jul 4;26:639. doi: 10.1186/s12891-025-08865-9 (PMC12231845; doi:10.1186/s12891-025-08865-9)
Supplement: Supplementary file 1 — Supplementary Material 1. [file 12891_2025_8865_MOESM1_ESM.docx]

Questionnaire

## Physical activity – retrospective UCLA activity scale

| **What was your physical activity level AT YOUR BEST CONDITION after the primary surgery?**  The 10 categories of physical activity load your hip increasingly from level 1 to 10. Consider your physical activity level when you were IN YOUR BEST CONDITION after the primary surgery. Please mark only one option |
| --- |
| ○ 1. Wholly inactive: dependent on others, cannot leave residence |
| ○ 2. Mostly inactive: restricted to minimal activities of daily living |
| ○ 3. Sometimes participate in mild activities, such as walking, limited housework, and limited shopping |
| ○ 4. Regularly participate in mild activities, such as walking, limited housework, and limited shopping |
| ○ 5. Sometimes participate in moderate activities, such as swimming and unlimited housework or shopping |
| ○ 6. Regularly participate in moderate activities, such as swimming and unlimited housework or shopping |
| ○ 7. Regularly participate in active events, such as bicycling |
| ○ 8. Regularly participate in very active events, such as hiking, bowling or golf |
| ○ 9. Sometimes participate in impact sports such as jogging, tennis, skiing, acrobatics, ballet, heavy labor, or backpacking |
| ○ 10. Regularly participate in impact sports such as jogging, tennis, skiing, acrobatics, ballet, heavy labor, or backpacking |

## Hip functioning and health related quality of life – retrospective hip disability and osteoarthritis outcome score (hoos)

| **Hip functionality AT YOUR BEST CONDITION after the primary surgery:**  This survey asks for your view about your hip. This information will help us keep track of how you feel about your hip and how well you are able to do your usual activities. Answer every question by ticking the appropriate box, only one box for each question. If you are uncertain about how to answer a question, please give the best answer you can. |
| --- |
| **Symptoms**  These questions should be answered thinking of your hip symptoms and difficulties when you were IN YOUR BEST CONDITION after the primary surgery  S1. Do you feel grinding, hear clicking or any other type of noise from your hip?  ○ Never ○ Rarely ○ Sometimes ○ Often ○ Always  S2. Difficulties spreading legs wide apart?  ○ None ○ Mild ○ Moderate ○ Severe ○ Extreme  S3. Difficulties to stride out when walking?  ○ None ○ Mild ○ Moderate ○ Severe ○ Extreme |
| **Stiffness**  The following questions concern the amount of joint stiffness you have experienced when you were IN YOUR BEST CONDITION after the primary surgery. Stiffness is a sensation of restriction or slowness in the ease with which you move your hip joint.  S4. How severe is your hip joint stiffness after first wakening in the morning?  ○ None ○ Mild ○ Moderate ○ Severe ○ Extreme  S5. How severe is your hip stiffness after sitting, lying or resting **later in the day?**  ○ None ○ Mild ○ Moderate ○ Severe ○ Extreme |
| 1. **Pain**   P1. How often is your hip painful?  ○ Never ○ Monthly ○ Weekly ○ Daily ○ Always  What amount of hip pain did you experience when you were IN YOUR BEST CONDITION after the primary surgery during the following activities?  P2. Straightening your hip fully  ○ None ○ Mild ○ Moderate ○ Severe ○ Extreme  P3. Bending your hip fully  ○ None ○ Mild ○ Moderate ○ Severe ○ Extreme  P4. Walking on a flat surface  ○ None ○ Mild ○ Moderate ○ Severe ○ Extreme  P5. Going up or down stairs  ○ None ○ Mild ○ Moderate ○ Severe ○ Extreme  P6. At night while in bed  ○ None ○ Mild ○ Moderate ○ Severe ○ Extreme  P7. Sitting or lying  ○ None ○ Mild ○ Moderate ○ Severe ○ Extreme  P8. Standing upright  ○ None ○ Mild ○ Moderate ○ Severe ○ Extreme  P9. Walking on a hard surface (asphalt, concrete, etc.)  ○ None ○ Mild ○ Moderate ○ Severe ○ Extreme  P10. Walking on an uneven surface  ○ None ○ Mild ○ Moderate ○ Severe ○ Extreme |
| 1. **Function, daily living**   The following questions concern your physical function. By this we mean your ability to move around and to look after yourself. For each of the following activities please indicate the degree of difficulty you experienced due to your hip when you were IN YOUR BEST CONDITION after the primary surgery  A1. Descending stairs  ○ None ○ Mild ○ Moderate ○ Severe ○ Extreme  A2. Ascending stairs  ○ None ○ Mild ○ Moderate ○ Severe ○ Extreme  A3. Rising from sitting  ○ None ○ Mild ○ Moderate ○ Severe ○ Extreme  A4. Standing  ○ None ○ Mild ○ Moderate ○ Severe ○ Extreme  A5. Bending to floor/pick up an object  ○ None ○ Mild ○ Moderate ○ Severe ○ Extreme  A6. Walking on flat surface  ○ None ○ Mild ○ Moderate ○ Severe ○ Extreme  A7. Getting in/out of car  ○ None ○ Mild ○ Moderate ○ Severe ○ Extreme  A8. Going shopping  ○ None ○ Mild ○ Moderate ○ Severe ○ Extreme  A9. Putting on socks/stockings  ○ None ○ Mild ○ Moderate ○ Severe ○ Extreme  A10. Rising from bed  ○ None ○ Mild ○ Moderate ○ Severe ○ Extreme  A11. Taking off socks/stockings  ○ None ○ Mild ○ Moderate ○ Severe ○ Extreme  A12. Lying in bed (turning over, maintaining hip position)  ○ None ○ Mild ○ Moderate ○ Severe ○ Extreme  A13. Getting in/out of bath  ○ None ○ Mild ○ Moderate ○ Severe ○ Extreme  A14. Sitting  ○ None ○ Mild ○ Moderate ○ Severe ○ Extreme  A15 Getting on/off toilet  ○ None ○ Mild ○ Moderate ○ Severe ○ Extreme  A16. Heavy domestic duties (moving heavy boxes, scrubbing floors, etc)  ○ None ○ Mild ○ Moderate ○ Severe ○ Extreme  A17. Light domestic duties (cooking, dusting, etc)  ○ None ○ Mild ○ Moderate ○ Severe ○ Extreme |
| 1. **Function, sports and recreational activities**   The following questions concern your physical function. The questions should be answered thinking of what degree of difficulty you experienced due to your hip when you were IN YOUR BEST CONDITION after the primary surgery  SP1. Squatting  ○ None ○ Mild ○ Moderate ○ Severe ○ Extreme  SP2. Running  ○ None ○ Mild ○ Moderate ○ Severe ○ Extreme  SP3. Twisting/pivoting on your injured hip  ○ None ○ Mild ○ Moderate ○ Severe ○ Extreme  SP4. Walking on uneven surface  ○ None ○ Mild ○ Moderate ○ Severe ○ Extreme |
| 1. **Quality of life**   Q1. How often are you aware of your hip problem?  ○ Never ○ Monthly ○ Weekly ○ Daily ○ Always  Q2. Have you modified your life style to avoid potentially damaging activities to your hip?  ○ Not at all ○ Mildly ○ Moderately ○Severly ○ Extremely  Q3. How much are you troubled with lack of confidence in your hip?  ○ Not at all ○ Mildly ○ Moderately ○Severly ○ Extremely  Q4. In general, how much difficulty do you have with your hip?  ○ None ○ Mild ○ Moderate ○ Severe ○ Extreme |

## health related quality of life – retrospective five-level-Euro-Qol five-dimension questionnaire (EQ-5D-5L)

| **Health related quality of life AT YOUR BEST CONDITION after the primary surgery:**  This survey asks for your view about your health. Under each heading, please tick the ONE box that best describes how your health was when you were IN YOUR BEST CONDITION after the primary surgery. |
| --- |
| **Mobility**  ○ I have no problems in walking about  ○ I have slight problems in walking about  ○ I have moderate problems in walking about  ○ I have severe problems in walking about  ○ I am unable to walk about |
| **Self-care**  ○ I have no problems washing or dressing myself  ○ I have slight problems washing or dressing myself  ○ I have moderate problems washing or dressing myself  ○ I have severe problems washing or dressing myself  ○ I am unable to wash or dress myself |
| **Usual activities (e.g. work, study, housework, family or leisure activities)**  ○ I have no problems doing my usual activities  ○ I have slight problems doing my usual activities  ○ I have moderate problems doing my usual activities  ○ I have severe problems doing my usual activities  ○ I am unable to do my usual activities |
| **Pain / discomfort**  ○ I have no pain or discomfort  ○ I have slight pain or discomfort  ○ I have moderate pain or discomfort  ○ I have severe pain or discomfort  ○ I have extreme pain or discomfort |
| **Anxiety / depression**  ○ I am not anxious or depressed  ○ I am slightly anxious or depressed  ○ I am moderately anxious or depressed  ○ I am severely anxious or depressed  ○ I am extremely anxious or depressed |

| We would like to know how good or bad your health was. This scale is numbered from 0 to 100. 100 means the best health you can imagine. 0 means the worst health you can imagine. Mark an X on the scale to indicate how your health was when you were IN YOUR BEST CONDITION after the primary surgery. |
| --- |
| 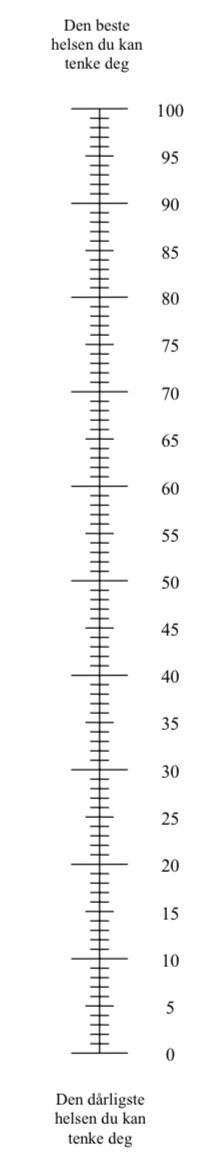 |
